# Uncovering Tacit Knowledge: A Pilot Study to Broaden the Concept of Knowledge in Knowledge Translation

**DOI:** 10.1186/1472-6963-11-198

**Published:** 2011-08-18

**Authors:** Anita R Kothari, Julia J Bickford, Nancy Edwards, Maureen J Dobbins, Mechthild Meyer

**Affiliations:** 1 Faculty of Health Sciences, University of Western Ontario, London, Canada; 2 Schulich School of Medicine and Dentistry, University of Western Ontario, London, Canada; 3 Faculty of Health Sciences and Faculty of Medicine, University of Ottawa, Ottawa, Canada; 4 Faculty of Health Sciences, McMaster University, Hamilton, Canada; 5 Population Health Improvement Research Network, University of Ottawa, Ottawa, Canada

## Abstract

**Background:**

All sectors in health care are being asked to focus on the knowledge-to-practice gap, or knowledge translation, to increase service effectiveness. A social interaction approach to knowledge translation assumes that research evidence becomes integrated with previously held knowledge, and practitioners build on and co-create knowledge through mutual interactions. Knowledge translation strategies for public health have not provided anticipated positive changes in evidence-based practice, possibly due in part to a narrow conceptualization of knowledge. More work is needed to understand the role of tacit knowledge in decision-making and practice. This pilot study examined how health practitioners applied tacit knowledge in public health program planning and implementation.

**Methods:**

This study used a narrative approach, where teams from two public health units in Ontario, Canada were conveniently selected. Respondents participated in individual interviews and focus groups at each site. Questions were designed to understand the role of tacit knowledge as it related to the program planning process. Data were analyzed through a combination of content analysis and thematic comparison.

**Results:**

The findings highlighted two major aspects of knowledge that arose: the use of tacit knowledge and the integration of tacit and explicit knowledge. Tacit knowledge included: past experiences, organization-specific knowledge, community contextual knowledge, and the recognition of the tacit knowledge of others. Explicit knowledge included: research literature, the Internet, popular magazines, formal assessments (surveys and interviews), legislation and regulations. Participants sometimes deliberately combined tacit and explicit knowledge sources in planning.

**Conclusions:**

This pilot demonstrated that front-line public health workers draw upon both tacit knowledge and explicit knowledge in their everyday lived reality. Further, tacit knowledge plays an important role in practitioners' interpretation and implementation of explicit research findings. This indicates a need to broaden the scope of knowledge translation to include other forms of knowledge beyond explicit knowledge acquired through research. Strategies that recognize and support the use of tacit knowledge, such as communities of practice or networks, may be important components of a comprehensive approach to knowledge translation. This study provides support for further investigation of the role of tacit knowledge in the planning and delivery of effective public health services.

## Background

Knowledge Translation (KT) has been defined by the Canadian Institutes for Health Research as "the exchange, synthesis and ethically-sound application of research findings within a complex set of interactions among researchers and knowledge users." [1]. Effective KT strategies are expected to lead to more effective health services and so contribute to the improvement of people's health. It is especially important to understand KT processes in the field of public health, where practitioners must operate in a complex environment, often under time constraints [2,3]. KT strategies have not, however, provided the positive changes in evidence-based public heath decision-making that had been anticipated [2].

One possible reason for this failure might be the overly narrow definition of knowledge. The bulk of KT research has focused on acquiring, assessing and applying research evidence in practice and policies. This lack of attention to a broader conceptualization of knowledge that goes beyond research findings [4] has consequently led to the development of KT strategies targeting only the use of research evidence. The role of other types of knowledge in the KT process has been downplayed in the literature, perhaps owing to the dominant epistemological position of logical empiricism that emphasizes causal relationships. In contrast, some researchers are beginning to turn to an interaction-based view of KT, in line with social constructivism. Social constructivism views knowledge, experience, realities and human understanding as socially constructed through interactions among people [5]. A social interaction perspective implies that users of research come together to discuss research findings and potential applications. During this conversation, they contextualize the research findings using their understanding of the communities and clients they serve, and the environment in which service delivery takes place [6]. In other words, research becomes integrated with previously held knowledge, and humans build on and create knowledge through their interactions with each other.

We use the previous work that has focused on understanding the best ways to promote the use of *research *findings, and build on this foundation by using a social interaction KT perspective for a more comprehensive understanding of *knowledge *translation in public health practice. Research literature is considered explicit knowledge, which can be described as knowledge that is often codified (written) and communicated through language. We know quite a bit about the use of research findings in public health settings. There is a need for relevant reviews of public health interventions [7,8] and ways to systematically capture the grey literature [9]. Other reported information needs include early reports on new health risks and emerging practices, synthesized information about established health risks and related programs, and evidence-based guidelines [10]. There is also a call for more active exchanges between front-line public health practitioners, researchers and decision-makers [2,11]. Public health decision-makers cite that barriers to the use of research findings include the time and ability to critically appraise studies, timeliness, availability and relevance of research, cost, credibility of researchers, policy climate, and implementation resources [8,10,12]. The value that an organization places on research use is likely to increase the use of systematic reviews in public health decision-making [12]. Complementing these empirical works are numerous conceptual frameworks for KT in public health practice, in which research literature is considered the information source of choice [3,13-15]. Researchers have implemented or are currently assessing KT strategies to encourage the uptake of research findings, with moderate success [11,16,13-19]. Devising innovative methods of translating research findings to practitioners continues to be a significant challenge [14,20].

In this pilot study we examined how health practitioners applied tacit knowledge in public health program planning and implementation. Our larger program of study aims to explore the role of tacit knowledge in public health practice and to identify potential knowledge exchange and implementation strategies that effectively incorporate tacit dimensions of knowledge. The term 'tacit knowledge' was first described by Polanyi, who stated, "...we can know more than we can tell" [21]. He proposed that tacit knowledge is difficult to communicate and is often acquired through practice and experience. Tacit knowledge is personal, practical and context specific, to the extent that even the knowledge holder may not be aware of its existence [22]. Terms like intuition, know-how, procedural knowledge, implicit knowledge, unarticulated knowledge, and practical or experiential knowledge have all been used to describe tacit knowledge [22]. According to Polanyi's concept, tacit knowledge is deeply related to skills and so is very difficult for the individual to articulate. Furthermore, tacit knowledge is not separable from explicit knowledge.

Tacit knowledge has been conceptualized differently by various disciplines and scholars, from which have sprung spirited debates untangling the various positions [23-25]. Consequently, there is no agreed upon definition of tacit knowledge. One train of thought sees tacit knowledge and explicit knowledge as two different entities, or at least as separate poles on a continuum, and that tacit knowledge is amenable to expression. The management literature, for example, identifies tacit knowledge as key to a firm's competitive advantage and innovation [26]. Considerable attention has been devoted to finding ways to "capture" this resource. Nonaka and Toyama [27,28] have expanded on Polanyi's concept by suggesting that there are both technical (i.e., skills- and experience-based) and cognitive aspects of tacit knowledge. The latter refers to beliefs, ideas and values, or mental models that are used for sense-making. Unlike Polanyi, Nonaka [27] suggests that there are patterns for creating knowledge and that a degree of tacit knowledge can be articulated. In this view of tacit knowledge, there is an emphasis on a socialization processes to support tacit knowledge sharing at the group level (note that Polanyi described tacit knowledge as deeply embedded at the individual level). The use of metaphors, analogies and stories can be used to convey tacit knowledge. We draw on Nonaka's concept of tacit knowledge, distinct from Polanyi's position, in our own work given its dominance and applied focus in the management literature. Specifically, we used a working definition of tacit knowledge from a review of the literature by McAdam and colleagues [24, p. 46]: who concluded that tacit knowledge is "knowledge-in-practice developed from direct experience and action; highly pragmatic and situation specific; subconsciously understood and applied; difficult to articulate; usually shared through interactive conversation and shared experience."

A handful of studies that focus on tacit knowledge have been carried out in the health domain. Herbig and colleagues [29] studied the response of nurses to hypothetical emergency situations. They found that while nurses who successfully addressed the situation used similar levels of explicit knowledge to those who were not successful, there was a marked difference in levels of tacit knowledge employed. They recommended instituting processes that promote the articulation of tacit knowledge at the individual and organizational level. Some researchers have emphasized the importance of tacit knowledge at the team level. Gabbay and le May [30] discovered the negotiated and co-constructed nature of knowledge in their study of the collective decision-making of nurses and general practitioners. Rather than drawing on research findings or explicit practice guidelines, study participants used collectively reinforced tacit guidelines based on experiences and interactions with one another in fluid communities of practice. Gabbay and le May suggest that discussions were important for sharing, testing, and internalizing these collective "mindlines". Friedman and colleagues [31], when examining the performance of multidisciplinary surgical teams, also came to the conclusion that teams' performances were dependent upon the unarticulated knowledge and understanding that occurred over time among team members. A case study of a multidisciplinary neuro-rehabilitation team found that standardized outcome results were discussed and interpreted with the aid of "embedded" or tacit knowledge held by the team based on clinical expertise and previous patients with similar results [32]. These studies highlight the interplay of tacit knowledge with explicit knowledge for skillful team practice.

An exploration of tacit knowledge is markedly missing from the KT and public health literature. A recent exception is Landry and colleagues [3] who draw from the organizational management literature to develop a conceptual framework for knowledge translation in public health. Their knowledge-value chain is a non-linear framework that outlines five capabilities necessary to manage knowledge, including: mapping acquisition, creation and destruction, integration and sharing/transfer, replication and protection, and performance innovation. Tacit knowledge is an integral aspect of these capabilities. We identified one empirical study in this area: Yoshioka-Maeda et al [33] explored the tacit knowledge of Japanese public health nurses and found that tacit knowledge was important for identifying community problems and then being able to respond quickly with needs-based programs. In this pilot study we examined how some Canadian health practitioners applied tacit knowledge in public health program planning and implementation, thus contributing to the limited knowledge in this area. This study also adds value to the larger KT landscape by bringing attention to a broader notion of "knowledge" than that represented by research findings.

## Methods

We used a narrative approach [34] for this pilot study, in which people make sense of their life experiences by telling stories [35,36]. This approach allowed the public health practitioners to retell and reconstruct the process of program planning in which they had recently been involved, and thus provided insight into the role that tacit knowledge played in the process [22]. The exploratory nature of the study led us to use a small sample and select a limited number of convenience sites. The research question was: how do public health practitioners use tacit knowledge for program planning?

Convenience sampling was used to select two public health units in Ontario, Canada; both units are situated in urban centres that also serve rural populations living around their urban cores. One team from each of the two public health units was invited to participate in the study. Inclusion criteria for participant teams were: they had to have at least three members, including one manager, and they had to have been involved in program planning in the last two years. Program planning involved the development or refinement of a health promotion initiative, such as a social marketing campaign to encourage breast cancer screening or a diabetes prevention cooking skills program for youth. Administrative staff were not considered eligible for participation in the study.

Eliciting tacit knowledge can be methodologically challenging given that such knowledge is difficult to articulate and is often embedded in routine practices. We adopted a two-step methods framework by Ambrosini and Bowman [22] with the specific purpose of eliciting tacit knowledge: individual interviews followed by a focus group at each site. Individual interviews provided participants with the opportunity to first generate narratives without cues from other team members. The interviewer encouraged participants to tell stories about the strategies that they had used during the program planning process.

In the focus groups we anticipated that collectively, team members would generate additional or different pieces of tacit knowledge about the program planning process. We developed a ten-item focus group guide (see Additional File 1: Focus Group Guide). Participants were asked to think about a recent program that all the team members present had been involved in planning (and that had been described in the individual interview) and to walk through the steps that were involved in the planning process. Participants also were asked to use markers and flip chart paper to construct a visual map of the steps involved in their program planning. According to Ambrosini and Bowman [22], causal or concept maps are representations of individuals' experience of reality that emphasize the causal connections or links between events. They are a means of eliciting tacit knowledge because they tend to focus on action and skills. As described by Hoffman [37], eliciting knowledge in this manner supports the individual to build up a representation of their domain knowledge. Causal maps place concepts in relation to one another and show interrelationships at a detailed, micro-level. The maps form a collective representation of reality and were built as a group activity.

The interviews and focus groups were digitally recorded and transcribed verbatim for analysis. Interviews were analyzed first to identify concepts that were then introduced during focus groups as the basis for the causal map [22]. Data were analyzed through a combination of content analysis and thematic comparison [38,39]. Three team members (JB, MM, and an additional researcher) coded two transcripts individually, looking for references to tacit and explicit knowledge. They then came together to compare the emergent codes and to construct a nineteen-item codebook, including definitions and examples for each code (see Additional File 2: Codebook for Qualitative Analysis). The entire team reviewed the codebook and definitions to ensure clarity. These three researchers went on to code the transcripts iteratively, allowing for on-going development of the codebook. For example, new codes emerged inductively and codes were also combined to reduce overlap. The coded transcripts were discussed, challenged and interpreted by the team as a whole. Analysis was aided by QSR N7. The causal maps were not analyzed because it was difficult to reconstruct at what point in the focus group an item had been added to the map, and when connections were made between the different items. We did not seek permission from the ethics boards to film the process because we had not considered the benefits of doing so when designing the study.

Each interviewer corrected and verified their transcripts by listening to the interview and reviewing the transcribed text. Transcripts were then shared with interview and focus group participants (member checking) to ensure accuracy, and corrections were noted. The team reduced threats to interpretation by having multiple researchers read and analyze the data independently and then come together for team analysis [40].

Informed consent was obtained from all study participants prior to participation in interviews and focus groups. This study was approved by the University of Ottawa ethics committee, as well as the University of Western Ontario ethics review board and the relevant Public Health Unit ethics committees.

## Results

There were five participants at the first site. This included one manager and four public health nurses, all of whom were female. The team was comprised of fairly experienced staff; the average time working in public health was 19.25 years with a range from seven to 30 years. The participants had been working on that particular team for an average of seven years with a range from five to 12 years. At the second site, five participants were interviewed individually. All five participants were nurses and all were female. The average period working in public health was 8.8 years with a range from 2 to 20 years. The average number of years on that particular team was 2.8 years with a range from six months to five years. Ten in-person individual interviews of approximately 30 to 45 minutes in length were conducted. Four participants attended the focus group at the first site, and four participated at the second site. Each group produced a causal map that stimulated a rich focus group discussion; the maps themselves were not analyzed (see Additional File 3: Causal Map from One Site) to see how the chain of events was sketched out and how elements that contributed to the pathway were added during the discussion.

The findings highlighted two major aspects of knowledge that emerged from the data: the use of tacit knowledge and the integration of tacit and explicit knowledge. Tacit knowledge in line with our working definition offered in the Background included: past experiences, organization-specific knowledge, community contextual knowledge, and the recognition of the tacit knowledge of others. A broad range of explicit knowledge drawn on by participants included: research literature, the Internet, popular magazines, formal assessments (surveys and interviews), and legislation and regulations.

### The Use of Tacit Knowledge in Public Health Program Planning

#### Past Experiences: Personal and Professional

Past experiences, both personal and professional, were a source of knowledge for program planning. When participants described the planning process for a program on how to effectively discipline adolescents, one participant explained that they had drawn on experiences with their own children to inform programs:

I think some of it is often influenced by the stage of life you're in yourself, right, too. If you've got young kids for you it's easier... so because I was at a stage of life where my son was a little older, I worked on the one [program] that was dealing with the adolescent stage of life and I found that much more interesting for me. I could pick lots of examples...scenarios that were common... that we were living through and 'Yeah, I've had to deal with this'. (Focus Group)

Personal experiences, such as going to a movie theatre, and observing food portions, was a form of tacit knowledge that shaped a public health program in one health unit. Observations of food portions that are commonly consumed might lead a public health program planner to consider interventions aimed at reducing such portion sizes or raising awareness about the impact.

...And also from, just what we see in the public. Like, I'm appalled when I go to a movie theatre and I see what people eat when they're sitting there. Like, especially the portion sizes just blow me away. (Focus Group)

All of the participants in this study had been active in public health planning for several years. As a result, they had accumulated experiential knowledge through their professional lives about strategies for successfully planning and implementing programs. For example, participants knew from past experience that in order to move a school health program forward, having face-to-face contact with school principals likely would be more successful than relying purely on the distribution of written material, like email. For the participants in this study, this assumption was based on a trial and error approach of what had worked in the past.

That's experience, because I think we found that so many things that you just hand out, they get lost in the shuffle. So you really have to do that personal contact with people, and we sent it out electronically to give them the 'heads up' that it was coming really...But electronically, like to go into a school in September is almost, it's ridiculous, because I think they probably open their inbox in September and there's like 2000 emails...I think all the [public health team's] ideas are from experience. I think so. It wasn't based on research...We know that works, right? And every school is different. (Focus Group)

Other examples of experiential knowledge included knowing who to contact for information, how to get buy-in from external partners, and when and how to assume leadership. Often the tacit knowledge acquired through personal and professional domains is combined in new ways - ways that might not conform to traditional evidence-based practice procedures - to meet the demands of a particular situation, as described below.

"It's a wealth of information that, that's held within our brain, that often is not tapped into unless you're asked or put in a position like I was, that I've got to start finding research to validate my, my proposal. And it's then you start realizing that we have a wealth of knowledge all around us. Um, we all, we all try to sort of be current with the issue and ah, the piece that we're lacking, or learning right now is the legislation piece. (Interview)

#### Organization-Specific Knowledge

Participants described the collectively held common knowledge that is taken for granted by employees of their organization. They described a type of organization-specific knowledge pertaining to core program elements. For example, each of the participants in one of the health units talked about the four components of school health. Although some participants could not remember where the components originated, they were all aware that they were integral to public health program planning.

We looked at the 4 components of comprehensive school health. They've been there forever, for a long time. I'm not even sure where they came from. (Focus Group)

Participants reflected on organizational practices, or a "way of doing things", that likely had not been captured in writing anywhere. They spoke about people in the organization (often using 'we') adopting practices that they would automatically apply and transfer when planning and implementing work on new issues and programs.

"We'd done one maybe two years previously around raves. And so there was already, sort of, for those would been around awhile [interruption by another participant: "It was a format"], an established way." (Focus Group)

This represents a type of programmatic-corporate knowledge that employees drew upon in the process of program refinement and redesign.

#### Community Contextual Knowledge

Local contextual knowledge is the intimate knowledge that one attains through familiarity with the local environment in which the public health program will be implemented. For one group of participants, local contextual knowledge was represented in the in-depth and nuanced knowledge they had about individual schools; they were familiar with the cycles and rhythms that took place over the day, month, and year; they could see what the students were eating for lunch; they heard out-of-breath students pant in the gym, and they understood the idiosyncrasies of particular school committees.

This contextual knowledge informed efforts to tailor programs to the distinctive needs of individual schools. For example, contextual knowledge about the differences in lunch suppliers for rural and urban schools was brought into consideration during decision-making about criteria for a particular healthy eating program:

The county schools don't have hot lunch suppliers like they do in the city. In the city they have a hot lunch, a company that will provide a package deal for a hot lunch, whereas the county schools don't have that. (Focus Group)

Being physically present at the location in which a program was being carried out allowed participants to gain a deeper understanding of program impacts through participant observation. A manager recounted a story about a young student who was involved in planning a school safety program. The manager described the lasting impression of hearing and watching this young child get involved in program planning. Local contextual knowledge allowed the manager to understand the impacts of public health programs and was a powerful complement to the knowledge gained through explicit research literature.

I can still see her face, she was just beaming; they were talking about physical activity and trying to come up with a winter wonderland kind of thing and they were talking about having a contest of building snow forts and she said, you know, 'don't you think that we should think about the safety factor of building snow forts' ...So I think, there is lots of literature to support this, but there's nothing quite like seeing it with your own eyes. (Focus Group)

#### Recognizing the Tacit Knowledge of Others

Another source of knowledge for participants was the knowledge held by others. Sometimes these were professionals on the program planning team, such as the dietitian or the librarian. Expert knowledge was also acquired by enlisting help from community members such as students, teachers, principals, custodians and parents when a program was being planned.

One participant explained that the expert knowledge from the dietitian on the team was utilized during the planning of a healthy eating program.

We showed the [nutritionists] what we had done. For example we were putting down serving sizes, because a lot of people don't know what a serving size is. Like a glass of juice is really only a half a cup, that's a serving, not a full cup. And so ...the [nutritionists] made sure it was accurate and ...gave us their feedback. (Focus Group)

Some participants described a shift in the way in which this type of knowledge was exchanged and accessed within public health over time. In particular, some participants explained the change from a silo model of practice to a more interdisciplinary model. For one participant, this shift in the model made expert knowledge more accessible. She commented that other teams within the health unit, health inspectors, and nutritionists were now "just phone calls away".

We've got a chronic disease and injury prevention team. We can go to them if there's physical activity, or if there's smoking issues; we can access the inspectors, infectious diseases, you know, whatever. We talk as nurses, we talk with the other professionals who are here too. So they're always just phone calls away. It's great...It used to be we never worked with health inspectors about anything, and we only had one nutritionist who was trying to meet the needs for everybody; now they're incorporated as members of different teams and there's a lot more, to go outside of your own silos. (Focus Group)

At one study site, much of the acknowledgement of external tacit knowledge related to how participants involved community partners in program planning and implementation processes. This knowledge included a set of community development skills: networking, seeking information from the target population and involving them in the planning process to facilitate program uptake. Working with partners with different interests, agendas and mandates, and knowing how to involve them in the planning process required both skills and contextual knowledge that seemed to be tacitly held.

Another participant commented on the key role that local experts play in program planning. This participant described students' knowledge as important to the success of programs.

I find that when you're working with, when you're trying to address a population, so for us it was the kids, and this is a JK to 6 school, the kids that are on this committee are grades 4 to grade 6, and so we asked the children, the students, 'how do you think they can get this across?' Would announcements be enough? No, because they don't listen to announcements. Would putting up posters be enough? No, that wouldn't be enough, we need to generate excitement; we need to do something that really makes it fun and they all said, 'kids love competition'. (Focus Group)

Parents, teachers, principals and custodians were also viewed as providing valuable expert knowledge that influenced public health program planning.

### The Use of Explicit Knowledge in Program Planning

The participants also described explicit knowledge as integral to planning throughout the interviews and focus groups. Explicit knowledge included research evidence, grey literature, the Internet, popular magazines, formal evaluations and assessments of programs by the public health unit, and government legislation and regulations. Some participants noted that explicit information was sometimes disregarded if it did not support the directions or priorities of those involved in planning. According to one participant, program planning is at times more influenced by who is around the table, what they feel comfortable doing, and how much energy they have than by explicit knowledge obtained through analyzing data or established 'best practices'.

### Integrating and Reconciling Tacit and Explicit Knowledge

Overlaps between explicit and tacit knowledge occurred when respondents described organizational structures and procedures, talked about the integration of information from actual data with experiential knowledge, or described ways in which they attempted to fulfill the health unit's mandate. Participants provided several examples in which tacit and explicit knowledge sources were deliberately combined in planning, as illustrated below:

You never have the full picture, one of the reasons we're going to be looking at our assumptions in that planning meeting is that there isn't enough data and we have to supplement the data with what we think we know through our experiences and sometimes even just what we see over and over on television. Bringing that to the table and giving it its proper weight but making it part of the whole, that whole picture that we're looking at. (Interview)

One participant described the process of drawing on multiple sources, beginning with explicit knowledge and then incorporating practical organizational and contextual knowledge when making decisions about program planning and priorities.

One, we'll have the literature, what the literature says. So we'll say ok, this is what we should be doing, can we do it? Then we look at very practical day-to-day realities. Do we have anyone to work with? Cause it, with this work we can't do it alone, there's too many people in ["X" location]. How many staff do we have? How much time can someone dedicate to this? What's the skill set of the people who'd be working on it? Do we have any resources? Is there any interest in the community? (Interview)

The data demonstrated that the combined use of explicit and tacit knowledge was a commonly cited occurrence.

## Discussion

This pilot study demonstrated how tacit knowledge, in addition to explicit knowledge, was drawn upon in the everyday lived reality of front-line public health workers. It might be argued that tacit knowledge even exerted a greater influence on program planning than research evidence. There is a growing understanding that "getting evidence into practice" is a complex process that involves different disciplinary approaches, beliefs, values and worldviews [41]. Even explicit evidence-based medicine rests on a foundation of implicit tacit knowledge [42]. In particular, there is a growing awareness of the different preferences for various types of knowledge in particular contexts. For example, Estabrooks et al [43] found many sources of practice knowledge utilized by nurses in tertiary-level hospitals in Alberta and Ontario. Through ethnographic interviews and participant observation they found that nurses relied on social interactions, experience, documents, and *a priori *knowledge [43]. The authors found that nurses frequently privilege and prefer experiential knowledge to more traditional formal sources (i.e. books, journals). Our findings agree with those of Rycroft-Malone et al. [44], who developed a taxonomy of knowledge sources including research, professional knowledge/clinical practice, local information, and patient experiences/preferences. Our methods (narrative approach, concept maps) and findings also move beyond these studies in that they demonstrate how expertise, experience and context are used as a tacit skill and a way of seeing the practice world [27,28], i.e., how tacit knowledge functions for the knower.

Participants in this study referred to tacit knowledge both at an individual and collective level. Other researchers have identified this additional layer of knowledge that is held collectively by health care teams [30-32]. These researchers also highlight the important element of time for accumulating shared understandings. Furthermore, in terms of teams, the findings from this pilot study suggest that a much wider range of stakeholders should be involved in a public health-based KT process. Community partners (school principals, teachers, students, community committees, police, etc) are involved in the public health planning process. While these stakeholders bring a wealth of tacit knowledge to public health practice, the involvement of these stakeholders in KT strategies has received limited attention. The community-based participatory research body of work provides an excellent starting point for working with community members, and readers are directed to Lencucha et al. [45] and Kothari and Armstrong [46] for detailed discussions about the relationship between this research approach and KT. Strategies that recognize and support the use of tacit knowledge, such as communities of practice or networks, may be important components of a comprehensive approach to KT. These strategies would support the perspective that tacit knowledge is conveyed through personal interactions and the construction of a shared understanding. It is difficult to determine from our study whether traditional group planning meetings are more in keeping with explicit knowledge sharing or whether they also function to support tacit knowledge, i.e., dialogue and shared understandings.

The findings also point to the many differing contexts (e.g., each school setting) faced by public health teams; this creates an interesting challenge in the integration of explicit and tacit knowledge that is distinct from much of the literature which assumes a common organizational context. This implies the importance of examining KT in situ or within the messy team-based contexts in which knowledge is negotiated, co-developed and played out. Examining KT by extracting individuals from the social and political contexts in which knowledge and evidence comes to be recognized and legitimated may lead to a superficial analysis. In situ research could reveal the factors or processes that are used to assess the validity of various sources of tacit knowledge (e.g., to distinguish between prejudice and fact).

To summarize, this preliminary work is novel in that it provides systematic data about tacit knowledge in the public health context, where variable amounts of literature-based intervention recommendations are available for planning. This study is also important for the broader KT research field, which tends to focus on individual clinicians or organizational-level supports; this study was designed to showcase the knowledge translation occurring among teams. In terms of study implications, the way in which explicit and tacit knowledge are integrated might be one of the most important aspects in the exploration of KT, and may shed light on new approaches to strengthening KT in public health. We might ask: where does tacit knowledge begin, and where and how does it overlap with explicit knowledge, if at all? What factors influence the interface between tacit and explicit knowledge and how do these vary during the program planning and evaluation cycle? This information can be used to determine when to encourage the appropriate use of tacit knowledge vis-à-vis explicit knowledge. For example, one might predict and then examine if tacit knowledge can support the contextual adaptation of explicit knowledge (e.g., research findings) so that they are relevant and applicable to local populations and conditions. The key message arising from this work is that for more successful KT initiatives we ought to consider the role of tacit knowledge in intervention design, implementation protocols and/or in understanding the underlying mechanisms related to knowledge creation, dissemination and utilization.

As this was an exploratory pilot study, the findings are not meant to be generalized to a larger population. In terms of limitations, the small sample size might have prevented a range of tacit knowledge experiences to emerge, and the self-reported data may not be as accurate as other forms of data collection. Also, some readers who use a different definition of tacit knowledge may question whether the findings align with their definition of tacit knowledge; this is an issue of scholarly debate and we invite further dialogue about the concept. Readers are reminded that our findings about tacit knowledge are contextually bound and pertain to a specific sense of tacit knowledge. We note that the findings were seen as credible by front-line practitioners [47]. The main strength of this study is that we used a theoretically informed narrative approach - asking public health practitioners to describe and collectively map their planning processes - to mitigate the effect of recall bias and to uncover the taken-for-granted nature of particular practices. The value of this study is as a springboard to better understanding the role of tacit knowledge in public health and to start building theory in the area. Future studies are called for to determine which types of knowledge are drawn upon in various circumstances. For instance, are tacit and explicit knowledge viewed differently at various decision-making levels for public health programs (i.e., front-line, managers, directors, medical officers of health)? Can we assume that other public health professionals (i.e. inspectors, nutritionists, health promotion workers, policy-analysts) view tacit and explicit knowledge in the same ways? The results of this study provide support for further investigation of the role of tacit knowledge in the planning and delivery of health services.

## Conclusions

This pilot study indicates a need to broaden the scope of knowledge translation to include other forms of knowledge beyond formal, explicit knowledge acquired through research. Tacit knowledge is multifaceted and drawn from individual professional experiences as well as through shared understandings that develop with team members (co-workers and stakeholders) over time. Further, tacit knowledge seems to play an important role in making sense of, and implementing, explicit information. The area is ripe for further research, and we have suggested a few avenues of inquiry. The role of tacit knowledge has important implications for knowledge translation strategies, which until now have emphasized needs related to acquiring, assessing and applying explicit research literature. Optimal KT strategies can lead to improved public health programs and services, and/or more effective delivery of such services.

## Competing interests

The authors declare that they have no competing interests.

## Authors' contributions

AK and NE conceived of the study, and AK, JB, and MM executed the research. All authors contributed to data analysis and interpretation. The first draft of the manuscript was written by AK. All authors provided important critical feedback on subsequent drafts of the manuscript, and approved the final manuscript.

## Pre-publication history

The pre-publication history for this paper can be accessed here:

http://www.biomedcentral.com/1472-6963/11/198/prepub

## Supplementary Material

Additional file 1 **Focus Group Guide**. Ten-item focus group guide designed to elicit additional or different pieces of tacit knowledge about the public health program planning process. Click here for file

Additional file 2 **Codebook for Qualitative Analysis**. Nineteen-item codebook developed to capture instances of tacit and explicit knowledge, with definitions and examples for each code. Click here for file

Additional file 3 **Causal Map from One Site**. Causal map produced by the participants of one site that sketched out connections between events and stimulated a rich focus group discussion. Click here for file

## References

[B1] Canadian Institutes of Health Research Knowledge translation strategy 2004-2009: innovation in action 2004 MR21-56/2004E-HTML: http://www.cihr-irsc.gc.ca/e/26574.html

[B2] Kiefer L Frank J Di Ruggiero E Dobbins M Manuel D Gully PR Mowat D Fostering evidence-based decision-making in Canada: examining the need for a Canadian population and public health evidence centre and research network Can J Public Health 2005 96 Suppl 3 I-1 19 15913085

[B3] Landry R Amara N Pablos-Mendes A Shademani R Gold I The knowledge-value chain: a conceptual framework for knowledge translation in health Bull World Health Org 2006 84 8 597 602 10.2471/BLT.06.031724 16917645 PMC2627427

[B4] Upshur REG VanDenKerkhof EG Goel V Meaning and measurement: an inclusive model of evidence in health care J Eval Clin Pract 2001 7 2 91 96 10.1046/j.1365-2753.2001.00279.x 11489034

[B5] Lincoln Y Guba E Denzin N, Lincoln Y Paradigmatic controversies, contradictions and emerging confluences Handbook of Qualitative Research 2000 2 CA: Thousand Oaks 163 188

[B6] Wenger E Communities of practice: learning, meaning, and identity 1998 Cambridge, England: Cambridge University Press

[B7] Frank J Di Ruggiero E Moloughney B "Think tank on the future of public health in Canada" Can J Public Health 2004 95 1 6 12 14768733 10.1007/BF03403625 PMC6975849

[B8] Ciliska D Hayward S Dobbins M Brunton G Underwood J Transferring public-health nursing research to health-system planning: assessing the relevance and accessibility of systematic reviews Can J Nurs Res 1999 31 1 23 36 10455585

[B9] O'Carroll PW Cahn MA Auston I Selden CR Information needs in public health and health policy: results of recent studies J Urban Health 1998 75 4 785 793 10.1007/BF02344508 9854240 PMC3456015

[B10] LaPelle NR Luckmann R Simpson EH Martin ER Identifying strategies to improve access to credible and relevant information for public health professionals: a qualitative study BMC Public Health 2006 6 1 89 102 10.1186/1471-2458-6-89 16597331 PMC1456961

[B11] Kothari A Edwards N Brajtman B Campbell N Hamel N Legault F Mill J Valaitis R Fostering interactions: the networking needs of community health nursing researchers and decision-makers Evidence & Policy 2005 1 3 291 30421863554

[B12] Dobbins M Cockerill R Barnsley J Factors affecting the utilization of systematic reviews J of Inter Tech of Health Care 2001 17 2 203 214 10.1017/S0266462300105069 11446132

[B13] Kothari A Birch S Charles C "Interaction" and research utilisation in health policies and programs: does it work? Health Policy 2005 71 1 117 125 10.1016/j.healthpol.2004.03.010 15563998

[B14] Dobbins M Ciliska D Cockerill R Barnsley J DiCenso A A framework for the dissemination and utilization of research for health-care policy and practice Online J Knowledge Synth Nurs 2002 E9 1 149 160 12439759

[B15] Kitson A Ahmed LB Harvey G Seers K Thompson DR From research to practice: one organizational model for promoting research-based practice J Adv Nurs 1996 23 3 430 440 10.1111/j.1365-2648.1996.tb00003.x 8655816

[B16] Dobbins M Thomas H O'Brien MA Duggan M Use of systematic reviews in the development of new provincial public health policies in Ontario J Inter Tech of Health Care 2004 20 4 399 404 10.1017/s0266462304001278 15609787

[B17] Dobbins M DeCorby K Twiddy T A knowledge transfer strategy for public health decision makers Worldviews Evid Based Nurs 2004 1 2 120 128 10.1111/j.1741-6787.2004.t01-1-04009.x 17129325

[B18] Thomas BH Ciliska D Dobbins M Micucci S A process for systematically reviewing the literature: providing the research evidence for public health nursing interventions Worldviews Evid Based Nurs 2004 1 3 176 184 10.1111/j.1524-475X.2004.04006.x 17163895

[B19] Edwards N Kothari A CHNET-Works! A networking infrastructure for community health nurse researchers and decision-makers Can J Nurs Res 2004 36 4 203 207 15739945

[B20] Bartunek JM Rynes SL Daft RL Across the Great Divide: Knowledge Creation and Transfer between Practitioners and Academics Acad Manag J 2001 44 2 340 355

[B21] Polanyi M The tacit dimension 1966 London, England: Doubleday & Co

[B22] Ambrosini V Bowman C Tacit knowledge: some suggestions for operationalization J Manag Stud 2001 38 6 811 829 10.1111/1467-6486.00260

[B23] Duguid P "The art of knowing": Social and tacit dimensions of knowledge and the limits of the community of practice Inf Society 2005 21 2 109 118 10.1080/01972240590925311

[B24] McAdam R Mason B McCrory J Exploring the dichotomies within the tacit knowledge literature: towards a process of tacit knowing in organizations J Knowledge Manag 2007 11 2 43 59 10.1108/13673270710738906

[B25] Yang L Knowledge, tacit knowledge and tacit knowledge sharing: brief summary of theoretical foundation Int Conf Manag and Service Sci 2009 MASS '09 1 5

[B26] Ambrosini V Bowman C Surfacing tacit sources of success Int Small Bus J 2008 26 4 403 431 10.1177/0266242608091172

[B27] Nonaka I A dynamic theory of organizational knowledge creation Org Sci 1994 5 1 14 37 10.1287/orsc.5.1.14

[B28] Nonaka I Toyama R A firm as a dialectical being: Towards a dynamic theory of a firm Indust and Corp Change 2002 11 5 995 1009 10.1093/icc/11.5.995

[B29] Herbig B Büssing A Ewert T The role of tacit knowledge in the work context of nursing J Adv Nurs 2001 34 5 687 695 10.1046/j.1365-2648.2001.01798.x 11380737

[B30] Gabbay J le May A Evidence based guidelines or collectively constructed "mindlines?" Ethnographic study of knowledge management in primary care BMJ 2004 329 7473 1013 1018 10.1136/bmj.329.7473.1013 15514347 PMC524553

[B31] Friedman LH Bernell SL The importance of team level tacit knowledge and related characteristics of high-performing health care teams Health Care Manag Rev 2006 31 3 223 230 10.1097/00004010-200607000-00008 16877890

[B32] Greenhalgh J Flynn R Long AF Tyson S Tacit and encoded knowledge in the use of standardised outcome measures in multidisciplinary team decision making: A case study of in-patient neurorehabilitation Soc Sci Med 2008 67 1 183 194 10.1016/j.socscimed.2008.03.006 18403078

[B33] Yoshioka-Maeda K Murashima S Asahara K Tacit knowledge of public health nurses in identifying community health problems and need for new services: a case study Int J Nurs Stud 2006 43 7 819 826 10.1016/j.ijnurstu.2005.11.001 16356503

[B34] McCormick C Storying stories: a narrative approach to in-depth interview conversations Int J Soc Res Methodol 2004 7 3 219 236 10.1080/13645570210166382

[B35] Mishler EG Models of narrative analysis: a typology J Narrat Life Hist 1995 5 2 87 123

[B36] Colombo M Reflexivity and narratives in action research: a discursive approach Forum Qualitative Sozialforschung/Forum: Qualitative Social Research 2003 4 2 Art. 9.

[B37] Hoffman RR Lintern G Ericsson KA, Charness N, Feltovich P, Hoffman R Eliciting and representing the knowledge of experts Cambridge handbook of expertise and expert performance 2006 New York, NY: Cambridge University Press 203 222

[B38] Pope C Ziebland S Mays N Qualitative research in health care: analysing qualitative data BMJ 2000 320 7227 114 116 10.1136/bmj.320.7227.114 10625273 PMC1117368

[B39] Mayring P Qualitative content analysis Forum Qualitative Sozialforschung/Forum: Qualitative Social Research 2000 1 2 Art. 20

[B40] Patton MQ Qualitative evaluation and research methods: 1990 2 Newbury Park, CA: Sage Publications

[B41] Speller V Wimbush E Morgan A Evidence-based health promotion practice: how to make it work Promot Educ 2005 12 1 15 20 10.1177/10253823050120010106x 15952275

[B42] Thornton T Tacit knowledge as the unifying factor in evidence based medicine and clinical judgment Philos Ethics Humanit Med 2006 1 1 2 11 10.1186/1747-5341-1-2 PMC1475611 16759426

[B43] Estabrooks C Rutakumwa W O'Leary K Profetto-McGrath J Milner M Levers M Scott-Findlay S Sources of practice knowledge among nurses Qual Health Res 2005 15 4 460 476 10.1177/1049732304273702 15761093

[B44] Rycroft-Malone J Seers K Titchen A Harvey G Kitson A McCormack B What counts as evidence in evidence-based practice? J Adv Nurs 2004 47 1 81 90 10.1111/j.1365-2648.2004.03068.x 15186471

[B45] Lencucha R Kothari A Hamel N Extending collaborations for knowledge translation: Lessons from the community-based participatory research literature Evidence & Policy 2010 6 1 61 7521863554

[B46] Kothari A Armstrong R Community-based knowledge translation: Unexplored opportunities Implement Sci 2011 in press 10.1186/1748-5908-6-59 PMC3127775 21645381

[B47] Kothari A Edwards N Bickford J Meyer M Estable A The role of tacit knowledge in public health: A pilot study The Canadian Public Health Association Conference 2007 Ottawa

