# Supplementary material for: Uncovering Tacit Knowledge: A Pilot Study to Broaden the Concept of Knowledge in Knowledge Translation
Source: BMC Health Serv Res. 2011 Aug 18;11:198. doi: 10.1186/1472-6963-11-198 (PMC3173304; doi:10.1186/1472-6963-11-198)
Supplement: Additional file 1 — Focus Group Guide. Ten-item focus group guide designed to elicit additional or different pieces of tacit knowledge about the public health program planning process. [file 1472-6963-11-198-S1.DOC]

**Additional File 1: Focus Group Guide**

1. Can you explain the goals/objectives of your team?

2. Tell me about your roles and relationships within the team, i.e., team make-up.

3. Can you describe how you decide which particular programs/campaigns will be run?

4. Tell me about a community program or campaign that your team has recently conducted.

5. Tell me a story about the planning that was involved in this program.

6. Please describe a problem/challenge you faced during the development of this program and what steps you took to overcome this problem.

7. Tell me about how you worked as a team to overcome this challenge – how did you brainstorm? Decide on courses of action? Etc.

8. Please describe strategies you used to help inform your decisions.

Probes for questions 5 – 8:

How does that happen?

How did you feel when that happened?

What causes that?

Who is involved?

What influences that?

Does everybody see it that way? Why, why not?

Do you usually take these steps?

What is similar, dissimilar from your usual way? Why?

9. How did you feel about the ‘solution’ to this challenge?

10. Take me through the story again and tell me how you would do things differently if you were faced with the same situation again.
