# Supplementary material for: Uncovering Tacit Knowledge: A Pilot Study to Broaden the Concept of Knowledge in Knowledge Translation
Source: BMC Health Serv Res. 2011 Aug 18;11:198. doi: 10.1186/1472-6963-11-198 (PMC3173304; doi:10.1186/1472-6963-11-198)
Supplement: Additional file 2 — Codebook for Qualitative Analysis. Nineteen-item codebook developed to capture instances of tacit and explicit knowledge, with definitions and examples for each code. [file 1472-6963-11-198-S2.DOC]

**Additional File 2: Codebook for Qualitative Analysis**

| **Types** | **Definitions, explanation** | **Tacit** | **Explicit** |
| --- | --- | --- | --- |
| Demographics | Type of degree, number of years:  Length of experience;  Being on the team  Position (management versus other) |  |  |
| Program descriptions | Any detail provided about programs; how did they describe their program; program name; did they talk about more than one program |  |  |
| Methods used | How did they develop the program; Procedures, approaches, methods mentioned e.g., in decision making; who plans, involvement, descriptions of steps involved |  |  |
| Decision-making points | Specific references to a decision made about a program, or program direction; moments; changes in program direction |  |  |
| Roles | Knowledge about roles of specific personnel that is used to get information, network; |  |  |
| Organization specific knowledge | Knowledge that people within the organization hold, or about an organization that is generally ‘known’;  Attempts to transfer knowledge from one team to another | Collectively held knowledge  Organizational knowledge: |  |
| Emotional knowledge | Gut feeling about something;  I know this, I have a hunch; NOT based on experience they are referring to | Emotional knowledge, gut feeling |  |
| Context | Information about the context where the program is being delivered that influences planning | Local contextual knowledge; knowing |  |
| Information sources | What sources were used; Criteria for assessing how reliable certain sources are, resource assessment; External sources for information, internal |  | Formal evaluation: interviews with principals  Using the librarian  Using articles  Using formal sources  Using internet |
| Creative thoughts, ideas | Spontaneous ideas, no knowledge where they came from, or why they were used | Creativity |  |
| Expert knowledge | Expert knowledge: People, community: directing you to another source of information |  | Local experts, drawing on them  Topic area experts: dieticians |
| Learnings | Learning, new insights that have led to change in the program, new decisions for program directions | Learning from the past |  |
| Group process | Reference made to how the team is working; Team dynamics, Group dynamics described; |  |  |
| Ownership: We versus I  WORD SEARCH | Use of pronouns: we, us, I, group ownership versus: | We knew |  |
| Application of knowledge for future program planning | Thoughts why something might be useful for something else as well; for planning programs in the future |  |  |
| Health, health promotion, theoretical knowledge | Assumed motivators for behavioural change, to obtain goals; theory behind behavioural change |  |  |
| Experience:  Professional experience  Personal experience | Knowledge that is based on previous experience | Transferring knowledge from other parts of their lives:  Somatic knowledge: seeing or observing something |  |
| Assumptions  [might overlap organizational knowledge] | Assumption that everybody knows the reasons for something and agrees;  Including assumptions about shared values e.g., Comments that imply that certain values are better than others | Collectively held |  |
| Assessment | Success indicators described, that were likely not intended or explicit;  those which also were explicit  What information are they using to assess success |  | Evaluation, reports |
