# Supplementary figures and images for: Uncovering Tacit Knowledge: A Pilot Study to Broaden the Concept of Knowledge in Knowledge Translation
Source: BMC Health Serv Res. 2011 Aug 18;11:198. doi: 10.1186/1472-6963-11-198 (PMC3173304; doi:10.1186/1472-6963-11-198)

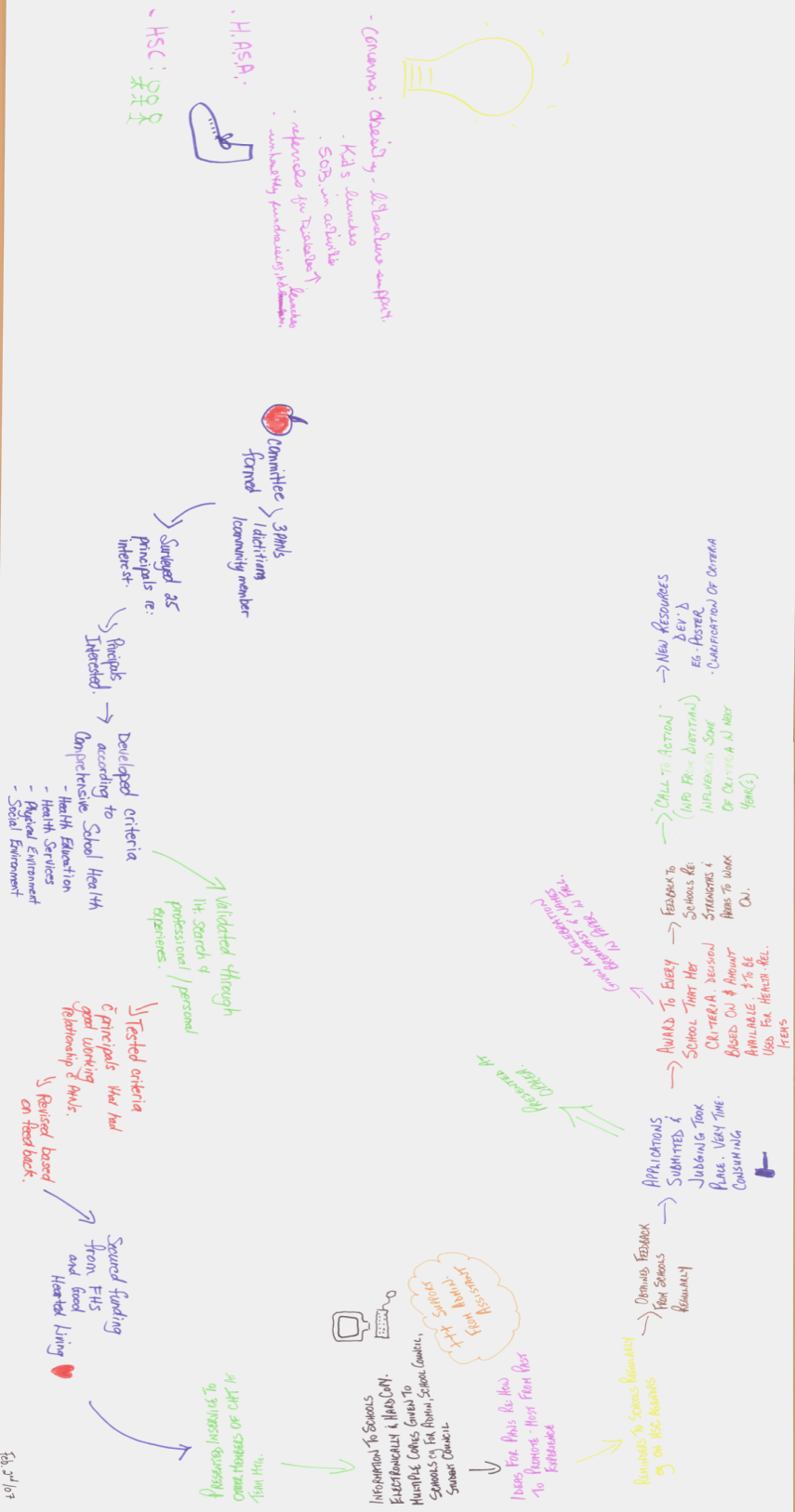
**Additional File 3: Causal Map from One Site**

Supplement: Additional file 3 — Causal Map from One Site. Causal map produced by the participants of one site that sketched out connections between events and stimulated a rich focus group discussion. [file 1472-6963-11-198-S3.DOC]
